# User reception of a simple online multisource feedback tool for residents

**DOI:** 10.1007/s40037-015-0173-0

**Published:** 2015-03-31

**Authors:** Lonneke Alofs, Jorike Huiskes, Maas Jan Heineman, Caroline Buis, Manon Horsman, Lars van der Plank, Olle ten Cate

**Affiliations:** 1 Center for Research and Development of Education, University Medical Center Utrecht, PO Box # 85500, 3508 GA Utrecht, The Netherlands; 2 Center of Evidence-Based Education, Academic Medical Center, University of Amsterdam, Amsterdam, The Netherlands; 3 Medical Center, Alkmaar, The Netherlands; 4 Family medicine practice, Utrecht, The Netherlands

**Keywords:** Multi-source feedback, Postgraduate medical education

## Abstract

Receiving feedback on daily clinical activities, in whatever form, is crucial for the development of clinical proficiency. Multisource or 360-degree feedback procedures have been recommended to include various co-workers as sources of feedback. In 2008, a web-based multisource feedback (MSF) tool for medical residents was developed at the University Medical Center Utrecht and launched nationally in the Netherlands and has been widely used since then. In 2012, an evaluation was carried out to collect opinions on its use, on the quality of the instrument and on its experienced effectiveness. We approached 408 residents and 59 residency programme directors with an anonymous online survey.

Completed surveys were received from 108 residents (26 %) and 22 programme directors (37 %). The tool was well received among the respondents and proved to be a simple, efficient and effective instrument to prepare for information-rich progress interviews of programme directors with their residents. Despite a relatively low response rate, indications were found for the effectiveness of MSF use at four levels of Kirkpatrick’s hierarchy based on user impressions: reaction, learning, behaviour change, and impact. This MSF tool, designed for effective formative feedback, was found to meet its purpose and was well received.

## Introduction

In less than 20 years’ time, feedback and assessment of medical trainees has become central to the thinking about professional development in the medical workplace. Miller’s famous pyramid of approaches to the assessment of medical trainees stimulated many educators to ponder on how valid formative and summative assessment should take place when trainees are immersed in the workplace, that is, at Miller’s ‘does’ level [1]. End-of-rotation examinations have often been replaced by series of short observations [2], teaching recommendations now usually include feedback procedures [3] and a multitude of observation-with-feedback approaches has enriched the clinical learning environment [4]

Specifically when learners must individually acquire skills by acting in practice, a sense of proficiency must be provided by the outside world, as learners generally have difficulty in evaluating themselves [5]. Informed self-assessment is considered an important skill for medical professionals [6] who may develop individually toward standards of competence. Without feedback, learners will have great difficulty in determining where they stand. While master-apprentice relationships in health care training were feasible and common until a few decades ago, current health care settings in industrialized countries have lost many of the natural moments for feedback in longitudinal preceptor-learner relationships [7]. Deliberate restructuring of workplace learning settings to incorporate guaranteed moments of feedback is therefore a logical and necessary development.

One of the tools that has emerged in health care training settings in the past decade is 360-degree or multisource feedback (MSF) [8], an approach derived from the business world.

MSF is the combined evaluation of a person by multiple individuals who have different working relationships with this person, through use of questionnaires and a compiled feedback report. For trainees, reviewers generally include peers, supervisors, other health professionals, and patients. Our study reports on the evaluation of an online MSF tool that has been applied in many residency programmes in the Netherlands since its inception in 2008.

## Aim and description of the MSF tool

In 2008, a multisource feedback tool (called ‘multisourcefeedback.nl’) was designed and launched at the University Medical Center Utrecht [9]. The aim of this application was to provide opportunities for programme directors of residency training throughout the Netherlands, to support the regular required progress interviews with individual residents with 360-degree feedback.

**Fig. 1:**
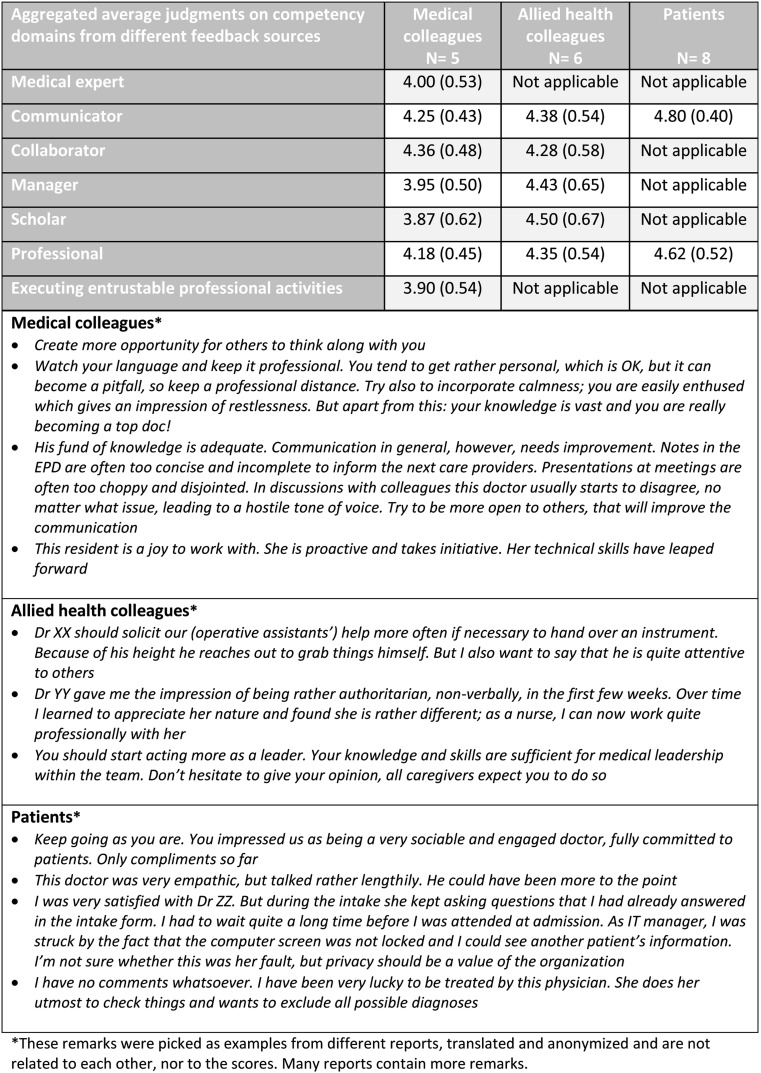
Sample MSF report (translated from Dutch)

The Utrecht instrument is simple to access and use [10, 11]. Any interested programme director who is registered with the Royal Dutch Medical Society can apply for an account. Next, a personal website is created that provides space to register his or her residents, including e-mail addresses, and the date for closure of the feedback process, usually 1–3 months later, when a report for each of them will be generated. The programme director starts the procedure by listing all residents who must receive MSF. The residents receive an e-mail requesting to provide e-mail addresses of multiple observers in three categories: medical colleagues (6 or more), other health care colleagues (6 or more), and patients (10 or more). The first two groups will receive an e-mail with a link to an MSF subsite that contains a questionnaire with space for narrative comments: ‘tops’ to stress positive observations, and ‘tips’ for improvement. Patients are asked to participate after a clinical encounter with the resident, in the hospital or after ambulatory care. Residents can observe the response process online and send automatic reminders, but they cannot identify or access individual responses. The procedure stops at a closure date, preset by the programme director; a report is then generated and is automatically sent to the resident and the programme director. All quantitative questionnaire data are summarized in a small table, categorized according to the CanMEDS framework used in the Netherlands [12], and followed by a long list of ‘tips’ and ‘tops’ as provided by the respondents. Whereas the source of comments remains confidential to the resident, the programme director may, for a limited period of time, identify respondents if desired. The programme director and the resident can discuss the report in the resident’s progress interview (Table 1, Fig. 1).

**Table 1:** MSF items, categorized in CanMEDS roles, and information sources

Item	Role	Information source		
I know this doctor well enough to evaluate his/her work		Physicians	Allied health	
Please give one or more recommendations so this doctor can function (even) better		Physicians	Allied health	Patients
Do you know this doctor from the clinic, the ward or both?		Physicians	Allied health	Patients
How long is the period you have been able to observe the resident?		Physicians	Allied health	
How many times did you see this doctor?				Patients
I would entrust this colleague with conducting a bad-news conversation		Physicians		
I would entrust this colleague with conducting a peer consultation		Physicians		
This doctor diagnoses patient problems effectively	Expert	Physicians		
This doctor deals with patient problems independently	Expert	Physicians		
This doctor weighs cost-benefit considerations about diagnostics and treatment	Expert	Physicians		
This doctor communicates adequately with patients and their family	Communicator	Physicians	Allied health	
This doctor communicates adequately with colleagues	Communicator	Physicians	Allied health	
This doctor is open to verbal and non-verbal reactions and emotions	Communicator	Physicians	Allied health	
This doctor presents a patient case accurately and briefly during a meeting	Communicator	Physicians		
This doctor gives clear and complete oral commands	Communicator		Allied health	
This doctor gives clear and complete written commands	Communicator		Allied health	
This doctor is clear and complete in written report	Communicator	Physicians	Allied health	
This doctor showed understanding for me	Communicator			Patients
This doctor showed empathy with me	Communicator			Patients
This doctor took me seriously	Communicator			Patients
This doctor listened carefully to me	Communicator			Patients
This doctor asked understandable questions	Communicator			Patients
This doctor gave clear explanations	Communicator			Patients
This doctor gave clear information about investigations and treatment	Communicator			Patients
This doctor verified whether I understood everything right	Communicator			Patients
This doctor hands over patient care effectively and carefully	Collaborator	Physicians	Allied health	
This doctor consults other caregivers in a timely manner	Collaborator	Physicians	Allied health	
This doctor appreciates and respects the knowledge and experience of others	Collaborator	Physicians	Allied health	
This doctor adheres to agreements and to an agreed policy	Collaborator	Physicians	Allied health	
This doctor takes responsibility for his/her own actions	Collaborator	Physicians	Allied health	
This doctor has found an adequate balance of patient care and personal development	Manager	Physicians		
This doctor coordinates the care of patients effectively	Manager	Physicians	Allied health	
This doctor is reachable and accessible	Manager	Physicians	Allied health	
This doctor organizes time effectively with the correct priorities	Manager	Physicians	Allied health	
This doctor timely passes on agreements and/or changes in plans	Manager		Allied health	
This doctor is willing and able to teach or train others	Scholar	Physicians	Allied health	
This doctor acts in an evidence-based manner if possible	Scholar	Physicians	Allied health	
This doctor provides feedback to others correctly	Scholar	Physicians	Allied health	
This doctor puts the interests of patients first during daily work	Professional	Physicians	Allied health	
This doctor takes patients privacy into account during physical examination	Professional	Physicians	Allied health	
This doctor knows the condition of the patient and the patient record information	Professional	Physicians	Allied health	
This doctor knows his/her own limitations and acts within them	Professional	Physicians	Allied health	
This doctor accepts feedback from others	Professional	Physicians	Allied health	
This doctor acts according to codes of ethical conduct	Professional	Physicians	Allied health	
I would recommend this doctor to my family and friends	Professional	Physicians		

In the 4 years between its launch in November 2008 and November 2012, *multisourcefeedback.nl* provided MSF reports for about 1000 residents in almost all speciality programmes across more than 40 hospitals, involving about 150 programme directors and over 15,000 individual respondents providing feedback. It was decided to evaluate the use of this tool to enable improvements if felt necessary. Using a questionnaire approach, we aimed to obtain an impression of its effect on all four Kirkpatrick levels of evaluation of a training intervention [13]: *reaction* (how did users experience the tool?); *learning* (did residents feel they learned about themselves from the feedback received?); *behaviour* (did the MSF report lead to a change in the behaviour of the resident?); *impact* (was there any indication that the resident’s environment benefited from this behaviour change, such as e.g. nursing and patients?) We prefer to use the word ‘impact’ instead of the original ‘results’ label as it is meant to reflect aspects such as improved production or quality, decreased costs, higher profits, decrease of adverse events [13], but not learning results.

## Methods

### Participants

Mid-2010 we had registered 82 programme directors, 524 residents, 6669 MSF questionnaire respondents from 18 speciality domains and within 32 teaching hospitals, among which three university centres, who had all used the tools at least once. For this study we included all 404 residents and 59 programme directors who had completed an MSF procedure between January 2010 and May 2012. The reason not to include earlier users was to avoid insufficient recall of the MSF procedure.

### The survey

We created two online surveys, one for residents and one for the programme directors. The initial surveys were designed by two authors (LA and OtC) and reviewed for clarity by a programme director (MJH) and anonymous MDs with trainee experience. Many questions were identical or slightly rephrased as they were relevant for both groups. The survey for residents included 44 questions, the one for programme directors 41 questions. Most were five-point Likert-type questions ranging from totally disagree—disagree—neutral—agree—totally agree, some had a three-point score (‘yes’, ‘no’ or ‘somewhat’) and some were open questions. The survey was distributed anonymously using the commercial tool SurveyMonkey^TM^.

### Analysis

As this was a fully descriptive study, no significance testing was done. All analyses were performed using Microsoft Excel. We interpreted the Likert scale answers as being an interval scale, and calculated a mean and standard deviation, but we added the percentages of respondents who agreed or totally agreed with a statement, given the debate about reporting on Likert scale data [14].

### Ethical approval

Approval to conduct this study was obtained from the Ethical Review Board of the Netherlands Association for Medical Education.

## Results

From the 404 residents we approached, 108 (27 %) responded by completing the full survey (27 % males, 72 % females, 1 % unspecified). The response rate among the postgraduate programme directors we approached was 37 % (22 out of 59; 68 % males, 32 % females). About half (46 %) of the responding residents had completed multiple MSF procedures, the other half only one; 64 % of the responding programme directors had held more than five progress interviews with the use of MSF.

Responding residents were from programmes of (in decreasing numbers): anaesthesiology (25), obstetrics/gynaecology (19), internal medicine (8), paediatrics (8), ear, nose and throat (6) and 14 other specialities (5 or less). Thirty-six residents (33 %) indicated they had not been able to include patients as respondents, often due to their speciality (anaesthesiology, radiology, hospital pharmacy). When asked in which programme year MSF is useful, all respondents mentioned multiple years (1: 65 %, 2: 74 %, 3: 78 %, 4: 71 %, 5: 61 %, 6: 35 %). Not all programmes have a 6-year course, which means that most residents generally consider MSF useful in all programme years. Considering the report, 38 % of the residents agreed or totally agreed that the numerical report is useful (3.0 on the Likert scale, SD 0 1.0); however, 89 % agreed or totally agreed that the open ‘tips’ and ‘tops’ were useful (4.3 on the Likert scale, SD 0.8).

Programme directors who responded were from obstetrics/gynaecology (4), paediatrics (4) internal medicine (3) rheumatology (2) and 8 other specialities (1). When asked in which programme year MSF is useful, all respondents mentioned multiple years, but certainly in the first year (1: 82 %, 2: 64 %, 3: 77 %, 4: 59 %, 5: 59 %, 6: 27 %). Programme directors find the first three programme years most suitable for MSF, and indicate that 1 or 2 times would be best (mean 1.7, range 1–3). Considering the report, 45 % of the programme directors agreed or totally agreed that the numerical report is useful (3.3 on the Likert scale, SD 0.9); however, 100 % agree or totally agreed that the open ‘tips’ and ‘tops’ were useful (4.7 on the Likert scale, SD 0.5). When asked how helpful, only 27 % agreed or totally agreed with the statement that the MSF report is sufficient in identifying poorly functioning residents (mean 2.9, SD 0.9). We asked both groups whether ‘adding self-assessment would be useful’, which was answered with (average) 3.0, SD (1.2) by residents and 4.5 (SD 0.7) by programme directors on a Likert scale, the latter group yielding this as a clear recommendation.

Table 2 shows the results of the remaining questions, grouped according to Kirkpatrick levels of evaluation outcomes of educational interventions [13].

**Table 2:** Survey item scores, categorised in levels of Kirkpatrick

Question (‘my/the resident’s’ reflect the two versions of the questionnaire)	Residents	Programme directors
	(*N* = 108)	(*N* = 22)
	Mean (SD)	Mean (SD)
*Kirkpatrick level 1 – REACTION*		
The multisourcefeedback.nl tool is user friendly	3.9 (0.9)	4.1 (0.8)
The final report is comprehensible and useful	3.7 (1.0)	4.0 (0.7)
The questions are generally applicable in my/the resident’s situation	3.4 (0.9)	3.7 (0.6)
Time between MSF procedure and progress interview (at least a month) is (1 = too short; 5 = too long)	3.0 (0.6)	3.1 (0.6)
The number of respondents to approach (6 medical colleagues, 6 allied health colleagues, 10 patients) was doable	3.0 (1.3)	3.7 (1.0)
It is good that the report is anonymous	3.9 (1.1)	3.9 (1.3)
The MSF reports yield an adequate impression of myself/of the resident	3.5 (0.9)	4.1 (0.5)
The MSF reports have added value compared with other sources of feedback	3.5 (1.0)	4.3 (0.5)
A progress interview using MSF leads to more concrete learning goals than when without MSF	3.0 (1.1)	3.7 (0.8)
*Kirkpatrick level 2 – LEARNING*		
My/the resident’s medical knowledge improved due to MSF	2.4 (0.9)	2.7 (0.9)
The latest MSF report yielded adequate points for my personal development	3.5 (1.0)	–
The latest MSF report taught me something about myself I did not know before	2.6 (1.0)	–
Working with colleagues improved due to the latest MSF	3.1 (0.9)	3.4 (0.8)
*Kirkpatrick level 3 – BEHAVIOUR*		
I think my/the resident’s performance improved due to the latest MSF	2.8 (1.0)	3.4 (0.8)
I think my/the resident’s professional attitude improved due to the latest MSF	3.2 (1.0)	3.7 (0.8)
The relationship with patients improved due to the latest MSF	2.8 (0.9)	3.2 (0.9)
Did latest the MSF procedure make you/the residents adapt your/their behaviour? Yes	17 %	30 %
Slightly	37 %	55 %
No	46 %	15 %
*Kirkpatrick level 4 – IMPACT*		
My/the resident’s medical colleagues benefited from positive changes in my/his or her performance due to the latest MSF	3.1 (0.9)	3.3 (1.0)
My/the resident’s allied health colleagues benefited from positive changes in my/his or her performance due to the latest MSF	3.2 (0.9)	3.4 (1.0)
Patients benefited from positive changes in my/his or her performance due to the latest MSF	3.0 (1.0)	3.4 (1.0)
The clinical care improved due to the latest MSF	3.0 (1.1)	3.2 (0.9)

Many residents used the opportunity to provide additional comments. Strengths of the MSF tool mentioned were (in decreasing frequency): the anonymous nature, the diversity of raters, the more complete picture received as opposed to regular feedback, the simplicity and efficiency of the procedure, the space for open comments and the fact that trainees were forced to ask for feedback and clinicians were forced to provide it. When asked for points of improvement, residents mentioned adding self-assessment, avoiding an anonymous nature, avoiding quantitative scores as they seem to discriminate little, the difficulty of recruiting patients as raters, the possibility of bias because trainees choose their own raters and the fact that the tool is less applicable for some specialities with little direct patient care. The anonymous nature, very often mentioned as a strength, was also mentioned as a weakness by some respondents.

Programme directors used the space for comments to particularly confirm the more complete picture received as opposed to regular feedback and the tool’s simplicity and efficiency. The option to ask self-assessment was mentioned as a point for improvement. Anonymity was also regarded by the programme directors as both a strength and as a point for improvement.

## Discussion

From the data we collected, and from the wide use of the instrument, we conclude that our MSF tool is well received. The MSF service offered by the University Medical Center Utrecht is fully voluntary. Multisource feedback is not an obligation for graduate medical programmes in the Netherlands and other tools such as electronic portfolio applications also provide such service.

This standalone MSF tool is regarded as an easy-to-use, very informative and widely applicable instrument, but in programmes with less direct patient contact such as radiology and anaesthesiology, patients as respondents are not usually included. This limits the breadth of feedback sources, but not necessarily the validity, as excluding patients does not exclude important observers in these specialities. The tool provides reports on CanMEDS competency domains and therefore aligns with the most dominant competency framework for postgraduate medical training worldwide, among which the Netherlands. As MSF is less suitable to build an impression of medical expertise than a knowledge or skills test, this role is only evaluated with few items and only from one source, in contrast with other roles; the same holds somewhat for the scholar role.

We framed our evaluation objectives according to the Kirkpatrick hierarchy. A true evaluation according to level 2, 3 and 4 of this hierarchy requires different data than we were able to collect. We would therefore like to stress that it is only our impression that the users of the tool perceived effects on these levels, which does not necessarily correlate with more objective effect measures. Reported satisfaction with education may not correlate with knowledge gain [15, 16].

If viewed from a Kirkpatrick hierarchy perspective, we conclude that programme directors clearly confirm beneficial effects on all levels: the MSF procedure is satisfactory, residents seem to learn about themselves, they observe adaptation of behaviour of residents and confirm beneficial effects on the resident’s environment. At all four levels, residents are also generally positive, but less so than their programme directors. Items with average Likert scores lower than 3.0 include: an increase in medical knowledge (the only item where programme directors also score lower than 3.0 on average), improvement of relationships with patients, insights about themselves, and performance improvement, which signifies that most residents do not observe beneficial changes on these items. Value ‘3.0’ strictly reflects a moderate agreement with a statement, but a conservative interpretation is that a lower mean than 3.0 does not signify a benefit.

If viewed from the perspective of Van der Vleuten’s five-item utility formula for assessment instruments [18], the utility of this MSF tool as a formative assessment instrument is high: it is well accepted, it is efficient and has low costs, and it appears to have visible educational impact. The reliability was not determined; this was considered less relevant for the formative purpose of the tool, as will be explained below. The validity, in the sense of adding relevant information to other sources, was considered a strength by the respondents. Lockyer has recently evaluated three dominant systems of multisource feedback, two in the UK and one in Canada and found support for their quality as measured as validity, reliability, equivalence, feasibility, catalytic effect and acceptability [17]. Our instrument has not been studied as rigorously and this first publication of experiences was focused on satisfaction and effects as perceived by residents and programme directors. We can fairly say that feasibility and acceptability were favourable according to both groups and the catalytic effect was present, at least according to the programme directors.

Our multisource feedback instrument is deliberately designed to provide personal, formative feedback to support the quality of developmental progress interviews during the course of training and we believe we have succeeded in providing this. From management levels, suggestions have been received to use the results for summative decision-making about residents. We do not recommend this, for several reasons. The anonymous and personal nature of the reports is highly appreciated as the most frequently mentioned strength. It enables respondents to feel safe to provide frank feedback. Even though some respondents stress the wish to disclose names we believe that respondents, knowing that their scores and comments can be used to take important decisions, will be less open in their response, which decreases the validity of the instrument. Furthermore, scores for decision-making require high reliability to avoid unfair decisions. While older studies have shown that more respondents per trainee are necessary to arrive at sufficiently reliable scores for summative conclusions [19, 20] a recent review by Donnon and colleagues reported a reasonable number of respondents to yield reproducible MSF scores [21]. and other conditions should be met, such as training of respondents, which can be an obstacle for wide implementation [10, 22]. Our approach aimed at providing an easy-to-use, highly informative but not high-stakes assessment instrument. The reason for this is not primarily concern about its reliability, but concern that the purpose of this instrument might become compromised if raters deliberately adapt their scoring or comments with such consequences in mind. We must not forget that peers are an important source of feedback and there is a serious risk that the mere presenting of MSF as a decision-making instrument will cause ‘deals’ among them.

Several respondents of our survey have suggested adding self-assessment to the tool. We had not included this in the design as we view self-assessment as a result of information from the outside world, for which multisource feedback is one strategy [6]. It is therefore not logical to aggregate scores for self-assessment with scores from other sources. It is possible to compare self-scores with MSF scores, but again, we acknowledge that the open comments prove to be the most informative part of the tool, not the scores received. Programmes directors can, at any moment, ask residents to score themselves on CanMEDS competencies, either before the MSF procedure, before the progress interview, or after the progress interview. It is however possible to add a separate option to self-assess, which is currently being considered.

Some respondents question the validity of responses provided by observers chosen by the residents themselves. Having a programme director pick and monitor responses of observers could yield more objective, or less biased responses, but would sharply increase the workload and decrease the practicality of the procedures. In addition, chosen peers for review may not necessarily decrease bias [23] as more remote colleagues may yield less informative feedback.

There are limitations to our investigation. One lies in the relatively low response rate in both groups. Being cognizant of the many requests for online surveys clinicians receive, we decided that a 27 and 37 % response rate was sufficient to be able to report, as we did not intend to test for significance and generalize from the findings to a large population. We acknowledge that the picture could be different if we had had a high response rate, but suspect that we would not arrive at very different conclusions.

A second limitation is that, by using Kirkpatrick’s hierarchy for effects of educational intervention, we report on judgment rather than objective findings for ‘learning’, ‘behaviour’ and ‘impact’. These should be considered proxies for the Kirkpatrick levels rather than accurate outcomes.

Finally, the benefits of our MSF procedure partly depend on how MSF reports are being used. The nature of the progress interview is an important factor and is highly dependent on the way programme directors take time, prepare the session by studying the report in advance and stimulate their residents to do the same. From research by Sargeant et al. [24] it is known that personal guidance in the interpretation of multisource feedback reports can increase its acceptance. In at least one hospital that we know of, an intermediate counsellor discusses the MSF reports with the residents to prepare for the progress interview with the programme director, which appears to be a valuable addition to the procedure.

Multisource feedback is becoming mainstream in modern residency programmes. The procedure we have described is a feasible, valued, low cost and non-bureaucratic approach that has clear potential to increase the quality of residency training.

## Essentials

- Multisource feedback, using the web and email-based tool ‘Multisourcefeedback.nl’ is well received by residents and programme directors;
- Providing multisource feedback is experienced as efficient, simple and effective when using this tool;
- We found indications of beneficial effects of the MSF tool at four levels of the Kirkpatrick hierarchy;
- Our tool is particularly constructed for formative feedback, and less targeted at high stakes summative evaluation.
